# Design, synthesis, anticancer evaluation and docking studies of novel 2-(1-isonicotinoyl-3-phenyl-1*H*-pyrazol-4-yl)-3-phenylthiazolidin-4-one derivatives as Aurora-A kinase inhibitors

**DOI:** 10.1186/s13065-022-00852-8

**Published:** 2022-08-17

**Authors:** Meenu Beniwal, Neelam Jain, Sandeep Jain, Navidha Aggarwal

**Affiliations:** 1 Department of Pharmaceutical Education & Research, Bhagat Phool Singh Mahila Vishwavidyalaya, Khanpur Kalan, Sonepat, Haryana 131301 India; 2 Department of Pharmaceutical Sciences, Guru Jambheshwar University of Science and Technology, Hisar, Haryana 125001 India; 3 MM College of Pharmacy, Maharishi Markandeshwar (Deemed to be University), Mullana, Ambala, Haryana 133207 India

**Keywords:** Aurora-A kinase, Pyrazole, Thiazolidin-4-one, Cell cycle arrest, MTT assay, Docking studies

## Abstract

**Introduction:**

Aurora-A kinase is associated with the Aurora kinase family which has been considered a striking anticancer target for the treatment of human cancers.

**Objective:**

To design, synthesize, anticancer evaluation, and docking studies of novel 2-(1-isonicotinoyl-3-phenyl-1*H*-pyrazol-4-yl)-3-phenylthiazolidin-4-one derivatives as Aurora-A Kinase inhibitors.

**Method:**

A total of 21 Pyrazole derivatives **P (1–21)** were synthesized by using the Vilsmeier Haack reagent which was characterized by FT-IR, ^1^H NMR, ^13^C NMR, and Mass spectroscopy. The synthesized derivatives were evaluated for their potential in vitro anticancer activity by MTT assay and Aurora-A kinase inhibition assay.

**Results:**

The cytotoxicity assay (MTT assay) showed that compound **P-6** exhibited potent cytotoxicity (IC_50_ = 0.37–0.44 μM) against two cancer (HCT 116 and MCF-7) cell lines, which were comparable to the standard compound, VX-680. Compound **P-6** also showed inhibition of Aurora-A kinase with an IC_50_ value of 0.11 ± 0.03 µM. A Docking study was done to compound **P-6** and **P-20** into the active site of Aurora A kinase, in order to get the probable binding model for further study.

**Conclusion:**

A series of 21 novel pyrazole derivatives **P(1–21)** were designed, synthesized, in vitro anticancer evaluation, and docking studies for Aurora A kinase inhibition. The results established that **P-6** is a prospective aspirant for the development of anticancer agents targeting Aurora-A kinase.

## Introduction

Cancer is a stage of disease in which a group of cells has an irritating and uncontrollable growth [1, 2]. Strategies to prevent cell division by affecting the mitotic spindle have long been the subject of growing research on the development of anti-cancer drugs [3, 4]. Chemotherapy remains a major problem in the treatment of cancer and related disorders.

Various therapeutic agents have been successfully tested and used to eradicate different types of cancer, but side effects have always been shown to be major problems. Furthermore, patient compliance is also compromised [5]. Undoubtedly, there is a great need to develop new powerful, and effective therapeutic agents with better efficacy and fewer adverse effects. Research is underway to tailor a rational approach to pharmaceutical innovation in which a new small molecule invention is designed and produced to inhibit particular proteins or abnormally expressed pathways in cancer cells. This approach, called targeted therapy, is now widely useful for discovering new goals and developing new cancer drugs.

Targeted therapies work by influencing the processes that control the growth, division, and spread of cancer cells, and the symptoms that cause natural death in cancer cells, such as monitoring normal cells when they are damaged or aged.

The development of new molecules that bind to targets of biological interest is a breakthrough in cancer research.

The knowledge of cell cycle mechanism and pathways of cancer pathogenesis has led to novel strategies and targets like Aurora Kinases, Topoisomerases, Check Point Kinases (CPK’s), Cyclin-Dependent Kinases (CDK’s), Oncogenic Human Papillomavirus (HPV), ABC transporters, etc. [6–11].

Aurora kinase is an essential regulator of cell cycle progression in cancer cells which suggests it is an attractive target for the development of anticancer drugs. Aurora kinases are a highly conserved family of serine/threonine protein kinases that play a key role in regulating many fundamental mitotic processes [12–16].

In mammals, three Aurora kinases are found: Aurora-A, Aurora-B, and Aurora-C. Auroras A and B are best known for their distinct roles in controlling mitosis, but the role of auroras C is still unclear. Aurora-A is concerned with the maturation and separation of centrosomes, the integration of bipolar spindles, and mitotic penetration, while Aurora B is essential for accurate chromosomal isolation and cytokinesis [17–21].

Therefore, besieged inhibition of Aurora-A kinase has developed into a striking therapeutic approach in the treatment of cancer. Aurora-A was first described in human neoplastic cell lines but was later discovered to be more uttered in an extremely wide range of human tumors, including primary colorectal carcinoma, breast, ovary, etc. [22].

**Fig. 1:**
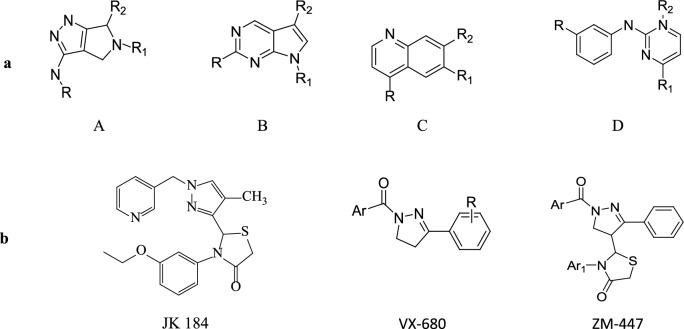
**a** Scaffolds found in Aurora-A kinase reported inhibitors (A) pyrrolo-pyrazole scaffold; (B) pyrrolo- pyrimidine scaffold; (C) quinoline scaffold; (D) diaminopyrimidin-2-anilino-e scaffold. **b** Examples of potent Aurora-A kinase inhibitors

The scaffolds of the renowned Aurora-A kinase inhibitors could also be categorized into four major groups labeled A-D, as given in Fig. 1. Based on the information, we have intended a series of new compounds by contributing an identical matrix through scaffolds A and D, assuming that the pyrazole group may be a common core of Aurora-A kinase inhibitors that inhibit Aurora-kinase A binding to its side adenosine triphosphate (ATP) [12, 23].

Encouraged by the various biological properties of thiazolidine-4-one and pyrazoles, we have attempted to synthesize the designated compounds using a hybridization approach in the expectation that the resulting novel compounds could react strongly with the Aurora-A kinase domain residues through hydrophobic interactions [13, 23–27].

**Fig. 2:**
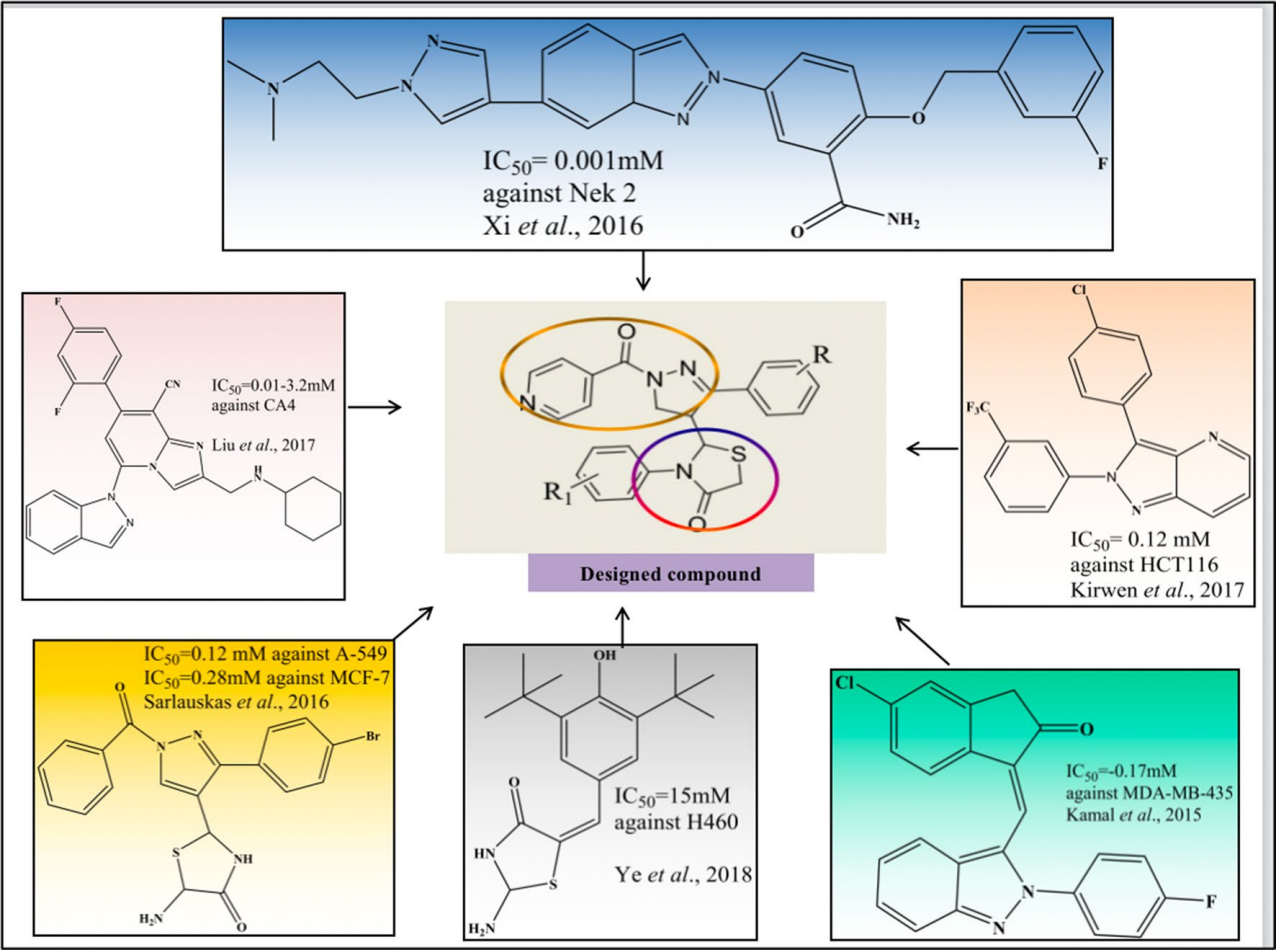
The design strategy for the generation of pyrazole scaffolds with thiazolidin-4-one derivatives

Most of the anticancer drugs used in the clinic come from heterocyclic. Pyrazole and thiazolidin-4-one nucleus have a scaffold in synthetic chemistry that has been successfully fueling researchers' interest in compounding various derivatives using a hybridization method for multiple chemotherapeutic activities involving cytotoxicity to cause apoptotic cell death in different cancer cell lines as shown in Fig. 2. Various conjugates possess pyrazole scaffolds in anticipation that potential novel compounds may react strongly with Aurora-A kinase domain residues through hydrophobic interactions [13, 23–27].

In summary, as a continuation of our study of antitumor compounds with the inhibitory activity of Aurora-A kinase, it is believed to be help form a series of 2-(1-isonicotinoyl-3-phenyl-1*H*-pyrazol-4-)yl)-3-phenylthiazolidin-4-one derivatives as a new class of Aurora-A kinase inhibitors are thought to have a synergistic effect on anticancer activity by inhibiting anti-Aurora A kinase inhibitors. Given the suggestions, we have directed our research to evaluate their anticancer activity in cell lines HCT-116 and MCF-7 to further investigate the initial mechanism of their role in the summary and further expand our research on antitumor compounds with inhibitory activity of Aurora-A kinase, which is considered useful to form a series of 2-(1-isonicotinoyl-3-phenyl-1*H*-pyrazol-4-yl)-3-phenylthiazolidin-4-one derivatives as a new class of Aurora- Kinases inhibitors as it may show a synergistic effect on anticancer activity by inhibition against Aurora-A kinase.

In light of the suggestions, we lead our research to evaluate their anti-cancer functions in cell lines of HCT-116 and MCF-7 to improve their study of their mechanism of mitosis.

## Result and discussion

### Chemistry

**Scheme 1:**
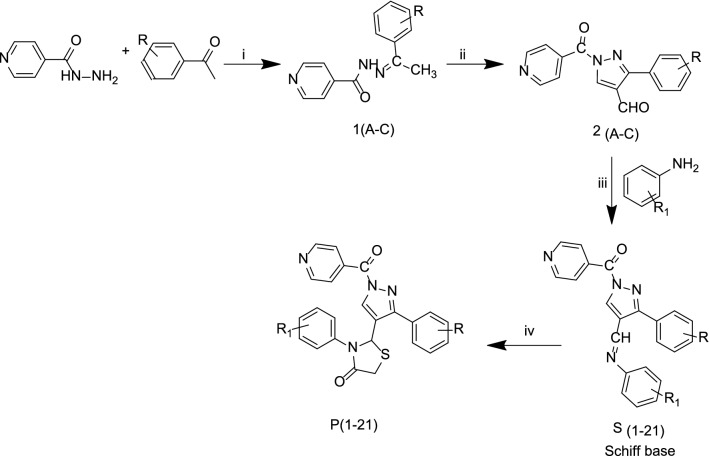
General synthesis of 2-(1-isonicotinoyl-3-phenyl-1H-pyrazol-4-yl)-3-phenylthiazolidin-4-one derivatives P (1–21). Reagents and conditions: (i) ethanol, glacial acetic acid, Conv.- reflux 30–40 min, MW-2–3 min (ii) DMF/POCl_3_, Conv.-reflux, 4–5 h, MW-3–4 min (iii) Conv.-reflux, 1-2 h, MW-3–4 min (iv) thioglycollic acid/DMF, Anhyd. ZnCl_2_, Conv.-1 h, MW-3–4 min

In current learning, we have reported syntheses of pyrazole derivatives **P (1–21)** by conventional (refluxing and stirring) as well as by green approach (microwave irradiation) [28, 29]. The synthesized derivatives **P (1–21)** were synthesized by condensation of Isoniazid with different acetophenone followed by Vilsmeier Haack reaction to form pyrazole-4-carbaldehyde 2(A-C) which was then treated with different anilines to get Schiff bases S (1–21). Schiff bases were further treated with thioglycolic acid in the presence of DMF and Zinc Chloride to form final compounds (Scheme 1). All the synthesized compounds were obtained in appreciable yield and other physicochemical properties are presented in Table 1 [28, 29].

**Table 1:** Physicochemical data of **2-(1-**isonicotinoyl-3-aryl-4,5-dihydro-1*H*-pyrazol-4-yl)-3-phenyl thiazolidin-4-one derivatives P (1–21)


Comp	R	R_1_	Mol. Wt	R_f_*	R_f_**	Conventional synthesis		Microwave-assisted synthesis			Melting point (°C)
						Reaction Time (hrs)	% yield	Microwave power (W)	Reaction time (min)	% yield	
**P-1**	4-NO_2_	H	473	0.60	0.58	1.0	68.2	300	2	89.2	244–246
**P-2**	4-NO_2_	4-NO_2_	520	0.37	0.50	1.0	67.3	300	2	87.4	266–268
**P-3**	4-NO_2_	2-NO_2_	520	0.20	0.32	1.0	60.5	300	2	83.1	282–284
**P-4**	4-NO_2_	4-Cl	507	0.23	0.24	1.0	56.9	300	2	80.0	286–288
**P-5**	4-NO_2_	3-Cl	507	0.69	0.64	1.0	59.2	300	2	75.8	274–276
**P-6**	4-NO_2_	3-OCH_3_	503	0.71	0.67	1.0	61.2	300	2	72.6	262–264
**P-7**	4-NO_2_	2-OCH_3_	503	0.66	0.62	1.0	67.4	300	2	93.4	250–252
**P-8**	4-OCH_3_	H	456	0.55	0.63	1.0	73.7	300	2	82.5	300–302
**P-9**	4-OCH_3_	4-NO_2_	503	0.53	0.71	1.0	68.9	300	2	76.3	308–310
**P-10**	4-OCH_3_	2-NO_2_	503	0.47	0.65	1.0	56.4	300	2	64.7	322–324
**P-11**	4-OCH_3_	4-Cl	490	0.35	0.70	1.0	59.2	300	2	68.8	318–320
**P-12**	4-OCH_3_	3-Cl	490	0.48	0.58	1.0	61.6	300	2	72.4	338–340
**P-13**	4-OCH_3_	3-OCH_3_	486	0.63	0.64	1.0	66.8	300	2	76.6	320–322
**P-14**	4-OCH_3_	2-OCH_3_	486	0.32	0.59	1.0	73.9	300	2	84.3	336–338
**P-15**	4-Br	H	504	0.70	0.65	1.0	66.8	300	2	84.7	256–258
**P-16**	4-Br	4-NO_2_	551	0.40	0.36	1.0	58.2	300	2	68.9	268–270
**P-17**	4-Br	2-NO_2_	551	0.21	0.35	1.0	61.2	300	2	72.0	252–254
**P-18**	4-Br	4-Cl	538	0.74	0.79	1.0	59.7	300	2	79.8	244–246
**P-19**	4-Br	3-Cl	538	0.70	0.74	1.0	64.2	300	2	72.3	248–250
**P-20**	4-Br	3-OCH_3_	535	0.60	0.65	1.0	61.3	300	2	83.2	252–254
**P-21**	4-Br	2-OCH_3_	535	0.28	0.32	1.0	58.4	300	2	69.1	260–262

TLC Mobile phase: *n-hexane: ethylacetate (4:6), **Ethylacetate: *n*-hexane: methanol (3:6:1)

The structures of compounds were elucidated by spectral data. The Infrared (IR) spectra of final compounds **P (1–21)** displayed absorption bands for C=N, C–H str (aromatic), CH-S str., C=C aromatic and C=O stretching at 1640–1650 cm^−1^, 3410–3120 cm^−1^, 2970–2920 cm^−1^, 1612–1563 cm^−1^, 2361–2349 cm^−1^ and 1720–1640 cm^−1^ respectively. IR spectra of compounds showed a strong and sharp band between 1660–1674 cm^−1^ and 1725–1714 cm^−1^ displayed carbonyl stretching of ═N–CO group present at pyrazole nucleus and carbonyl stretching of thiazolidinone nucleus, respectively. The presence of band at 1500 cm^−1^ and 1325 cm^−1^ displayed NO_2_ asymmetric and symmetric stretching respectively supported the presence of the nitro group in the structure of Comp. P-1, P-2, P-3, P-4, P-5, P-6, P-7, P-9, P-10, P-16 & P-17. The IR spectra of Comp. P-15, P-16, P-17, P-18, P-19, P-20 & P-21 showed stretching around 630 cm^−1^ depicting the presence of Bromo substituted phenyl ring in their structure. The presence of the band at 1222–1160 cm^−1^ displayed asymmetric C–O–C stretching vibrations whereas the band at 1099–1090 cm^−1^ displayed symmetric C–O–C stretching supported the presence of methoxy group in the structure of Comp. P-6, P-7, P-8, P-9, P-10, P-11, P-12, P-13, P-14, P-20 & P-21. The IR spectra of Comp. P-4, P-5, P-11, P-12, P-18, and P-19 showed a characteristic strong band at 720–664 cm^−1^ which was assigned to C–Cl stretching vibrations.

The ^1^H-NMR spectra (500 MHz, DMSO) of the compounds had a doublet around 8.06–7.96 ppm (J = 6–8 Hz) and 4.6–3.43 ppm (J = 12–16 Hz) for isonicotine hydrazide and thiazolidinone protons respectively. The chemical shift for the proton = CH– of the pyrazole ring and –CH– of the thiazolidinone ring was observed as a singlet between 8.38 and 8.55 ppm and 5.82–5.89 ppm respectively [28, 29]. The ^13^C-NMR spectra (500 MHz, CDCl_3_) of the compounds displayed peaks for pyridine, pyrazole, and thiazolidin-4-one at 149.8–122.8, 151.8–71.1, 47.8–34 respectively [30].

### Biological evaluation

#### Cytotoxicity assay

MTT assay was engaged to evaluate the anticancer activity of synthesized compounds P(1–21) against various human cancer cell lines (breast cancer- MCF-7 and colorectal carcinoma- HCT116) [31–34]. As illustrated in Table 2, the active analogs showed a noteworthy potential antiproliferative activity against cancer cell lines. Among these derivatives, Compounds **P-6** and **P-20** exhibited the highest cytotoxicity activity (IC_50_ = 0.39–0.56 µM). Particularly, it was noticed that compound **P-6** is slightly more active than compound **P-20** having IC_50_ = 0.37 ± 0.15 µM for HCT116 and IC_50_ = 0.44 ± 0.06 µM for MCF-7, comparable to the positive control VX-680 having IC_50_ = 0.32 ± 0.05 µM for HCT116 and IC_50_ = 0.40 ± 0.03 µM for MCF-7 cells respectively.

**Table 2:** Inhibition (IC_50_) of HCT116 and MCF-7 cell lines and inhibition of Aurora-A kinase by compounds P (1–21)

Comp	IC_50_ ± SD (µM)		
	HCT-116^a^	MCF-7^a^	Aurora- A^b^
**P-1**	2.30 ± 0.14	4.40 ± 0.18	2.34 ± 0.36
**P-2**	3.52 ± 0.33	3.26 ± 0.31	3.57 ± 0.35
**P-3**	4.47 ± 0.45	4.49 ± 0.42	7.25 ± 0.80
**P-4**	1.35 ± 0.07	2.55 ± 0.07	1.33 ± 0.15
**P-5**	3.88 ± 0.16	4.01 ± 0.10	4.79 ± 0.83
**P-6**	0.37 ± 0.15	0.44 ± 0.06	0.18 ± 0.05
**P-7**	0.62 ± 0.03	0.88 ± 0.03	0.42 ± 0.12
**P-8**	5.35 ± 0.27	6.08 ± 0.24	7.32 ± 0.69
**P-9**	1.45 ± 0.18	1.62 ± 0.05	1.45 ± 0.05
**P-10**	0.78 ± 0.04	1.03 ± 0.11	0.65 ± 0.09
**P-11**	2.54 ± 0.20	2.53 ± 0.19	2.78 ± 0.14
**P-12**	4.06 ± 0.38	4.35 ± 0.47	5.59 ± 0.37
**P-13**	5.87 ± 0.42	8.21 ± 0.35	12.37 ± 0.85
**P-14**	4.08 ± 0.31	5.30 ± 0.62	5.52 ± 0.55
**P-15**	2.18 ± 0.15	2.34 ± 0.27	2.10 ± 0.13
**P-16**	2.01 ± 0.11	2.25 ± 0.25	2.10 ± 0.15
**P-17**	2.92 ± 0.26	3.24 ± 0.13	3.04 ± 0.48
**P-18**	1.52 ± 0.09	1.77 ± 0.15	1.62 ± 0.11
**P-19**	1.02 ± 0.12	1.21 ± 0.05	0.79 ± 0.07
**P-20**	0.42 ± 0.08	0.56 ± 0.06	0.22 ± 0.08
**P-21**	0.97 ± 0.10	1.10 ± 0.08	0.79 ± 0.07
**VX-680 (**Tozasertib)	0.30 ± 0.03	0.38 ± 0.02	0.13 ± 0.01

^a^Inhibition of growth of tumor cell lines

^b^Inhibition of Aurora-A kinase

**Fig. 3:**
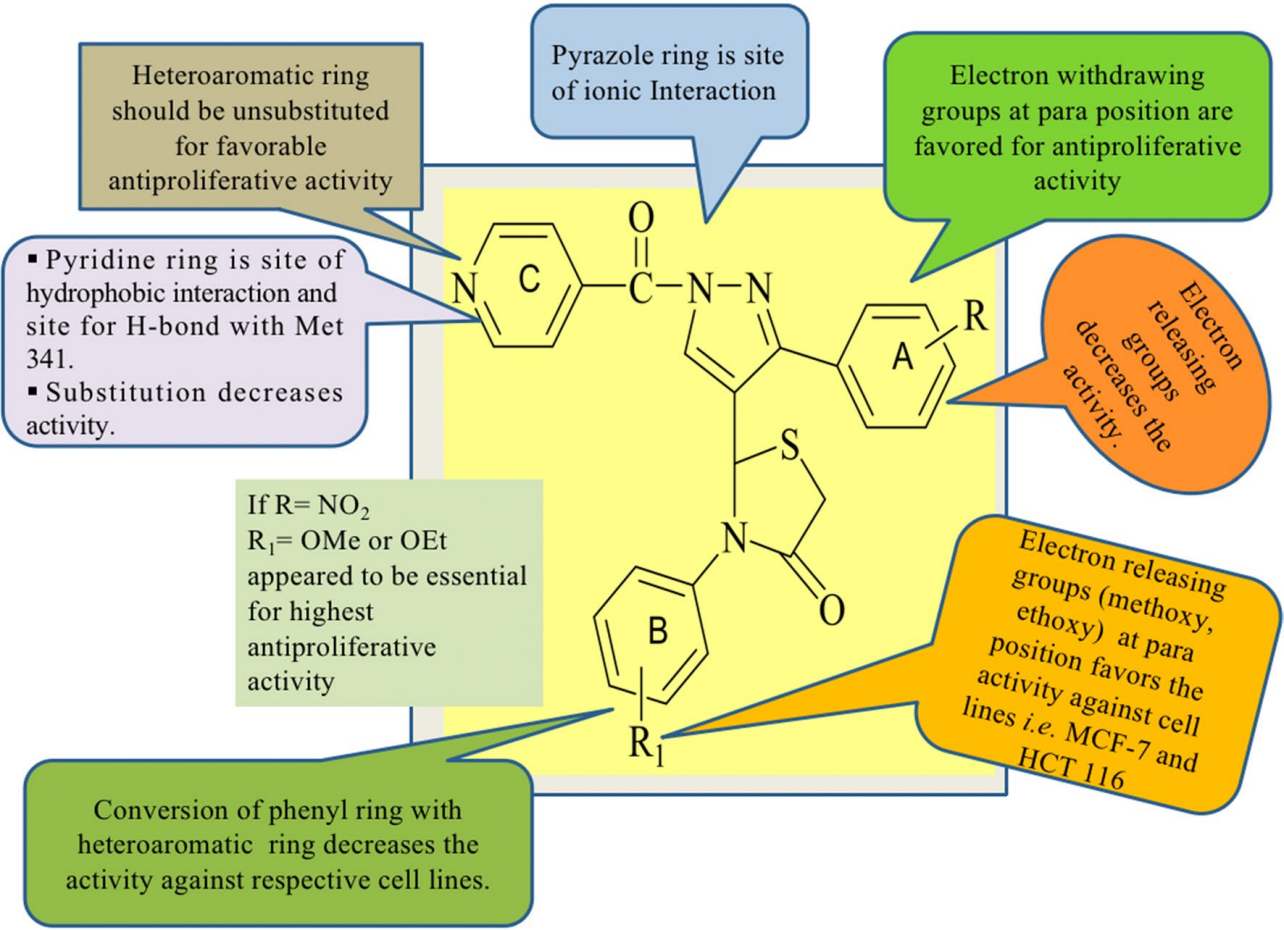
SAR of cytotoxicity of compounds **P(1–21)**

Results illustrated in Table 3 concluded that the activity of the tested compounds may be correlated to structural variation and modifications as shown in Fig. 3.

**Table 3:** Binding energy (Affinity) estimated by AutoDock Vina (PDB: 2bmc)


Comp	R	R_1_	Affinity (kcal/mol)	Log p	HBD	HBA	PSA	MR
P-6	**NO**_**2**_	**4-OCH**_**3**_	− 16.7	2.71	0	7	118.24	133.33
P-20	**Br**	**4-OCH**_**3**_	− 14.8	3.55	0	5	75.10	134.64
VX-680			− 25.6	4..87	3	7	49.46	136.36

By investigating the variation in the selectivity of the tested compounds over two cell lines, it was revealed that different substituents on the B-ring led to different antitumor activities. Among the compounds, the compounds with *para*-substituted A ring with electron- withdrawing substituents are of better antitumor activity compared to those with electron-donating substituents, the potency is ordered as NO_2_ > Br > Cl > H > Me > MeO whereas substitution of B ring at para position with the electron-donating group have improved antiproliferative activity, whereas electron-withdrawing groups (Cl, Br) had minor effects. Among all the compounds, **P-6** with the *p*-NO_2_ group on the A-ring and *p*-OCH_3_ on the B-ring respectively led to a noteworthy best activity.

To examine whether the compounds inhibit Aurora-A kinase, we screened compounds **P (1–21)** against the Aurora-A kinase via an enzyme-coupled continuous spectrophotometric assay, and the results were summarized in Table 2. As described in Table 3**,** compounds **P-6** and **P-20** showed the most compelling inhibitory activity with IC_50_ of 0.18 ± 0.05 µM and 0.22 ± 0.08 µM (positive control VX-680 with an IC_50_ of 0.11 ± 0.03 µM for Aurora-A kinase) respectively [35, 36].

The effects of Aurora-A kinase inhibitory activity of the tested compounds are consistent with the structure–activity relationships (SAR) of their antitumor activity. This has shown that the potent antitumor activity of synthetic compounds may be associated with their Aurora-A kinase inhibitory activity.

### Docking study

**Fig. 4:**
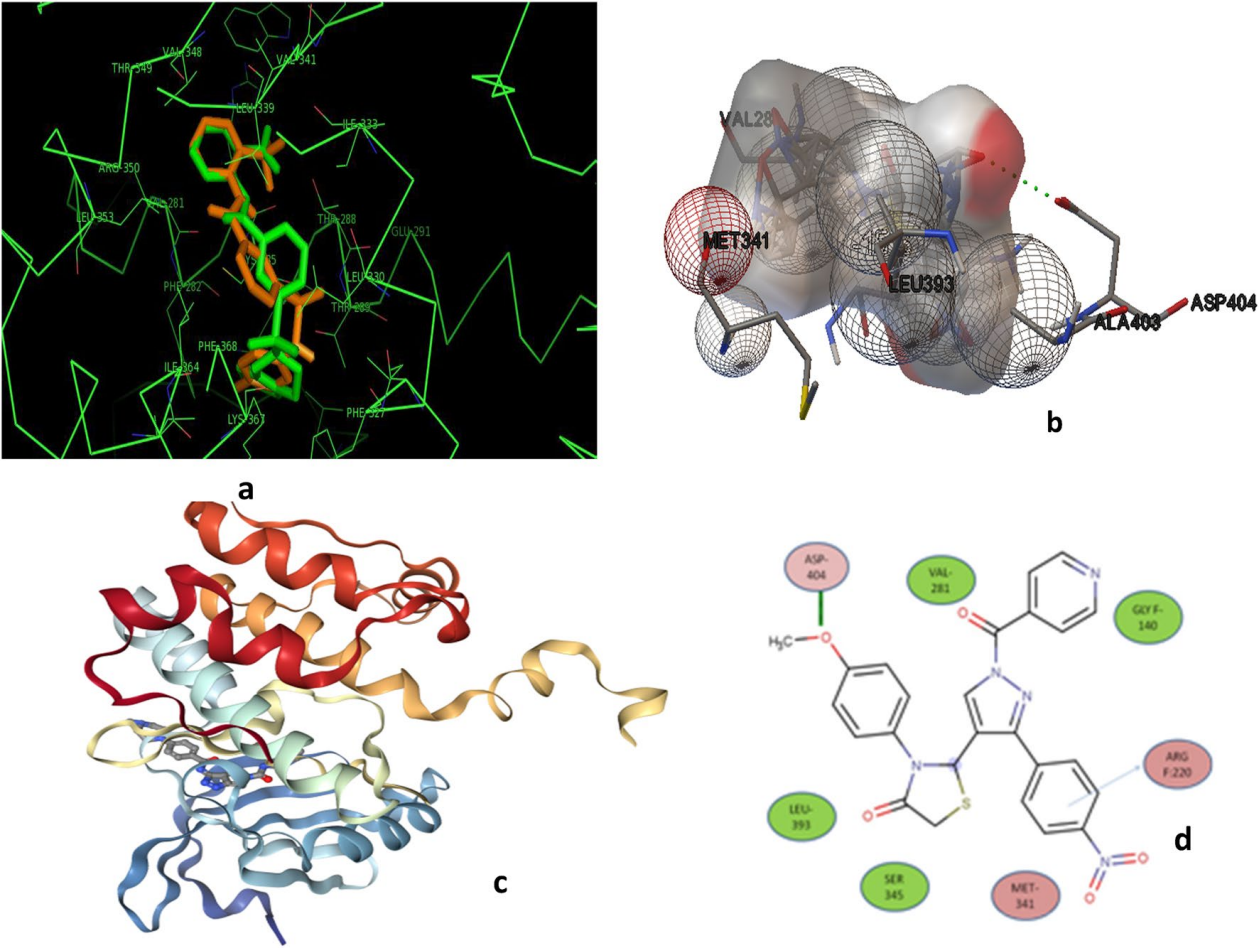
**a** Overlay of compound **P-6** (orange) with Aurora-A co-crystallized ligand (Pdb-2bmc). **b** Docked surface of ligand **P-6** with Aurora-A complex. **c** Surface model structure of compound **P-6** with Aurora A complex. **d** 2D ligand interaction diagram of compound **P-6** with Aurora-A using Discovery Studio program with essential amino acid residues at the binding site are tagged in circles. The purple circles show the amino acids which participate in hydrogen bonding, and electrostatic or polar interactions and the green circles show the amino acids which participate in the Vander Waals interactions

Docking studies were performed to evaluate the correlation and binding interactions of the integrated molecules in the active Aurora A kinase (PDB: 2bmc) [25, 37] using Auto Dock vina and Auto Dock tools installed in windows 7 [38, 39]. The docking protocol was tested with co-crystallized ligand in the active site of Aurora-A kinase and the resulting binding shape was compared with that of the co-crystallized ligand, VX-680 [36]. Designed compounds, **P (1–21)** are incorporated into the active site of Aurora kinase A. Both have a positive binding to the binding site as determined by the free binding force (ΔG) and the preferred docking binding mode (Table 3). Based on very low free bond strength and dock interaction in the binding area, the P-6 and P-20 compounds were also analyzed in detail by PyMOL. The compound overlay of P-6 and PDB ligand showed that the selected compound has the same binding pattern in the protein binding structure as that of the co-crystallized ligand and thus has the same type of binding (Fig. 4). The compound coverage of P-6 and PDB ligand helped us to predict that P-6 may act as a potent Aurora kinase A inhibitor.

The interaction of ligands **P-6** and **P-20**, VX-680 (positive control) with the receptor in terms of docking score (binding energy) was depicted in Table 3.

The binding bond of compound P-6 and Aurora-A kinase are shown in Fig. 4. Compound **P-6** is bonded to the ATP binding site of Aurora A kinase using hydrophobic bonding and binding is stabilized by hydrogen bond. The oxygen atom of the OCH_3_ group formed a single hydrogen bond with the amino hydrogen of Asp-404 (bond length: 1.79 Å; bond angle: 153.9). An enzyme surface model was shown in Fig. 4b, which showed that the molecule was neatly placed inside an active pocket. This effect of molecular docking and biological assay data suggested that compound **P-6** may be a potential inhibitor of Aurora-A kinase.

## Conclusion

In conclusion, a series of 2-(1-isonicotinoyl-3-phenyl-1*H*-pyrazol-4-yl)-3-phenylthiazolidin-4-one are synthesized and evaluated for their in vitro antitumor activity by MTT assay against HTC-116, MCF-7 cells lines and aurora kinase inhibitory activity. Amongst all compounds, **P-6** showed the most potent inhibitory activity which inhibited the growth of HTC-116 and MCF-7 cell lines (IC_50_ values of 0.37 ± 0.15 µM and 0.44 ± 0.06 µM) and inhibited the Aurora-A kinase with IC_50_ of 0.18 ± 0.05 µM, which was comparable to the positive control VX-680 (Tozasertib). Molecular docking was further performed to study the inhibitory Aurora-A kinase interactions. The data of this work might be helpful for the design and synthesis of a leading compound **P-6** toward the development of a new therapeutic agent to fight against cancer. The vast amount of current research on pyrazole has proven to be an effective aid in the development of new anticancer agents.

## Experimental

### Material and methods

All chemicals were procured from SD Fine Chem Co. Ltd and used in experiments. The melting point has been identified in the Bouchi oil melting point apparatus and has not been corrected. The microwave-assisted reactions were carried out in an HPL 2300 ET domestic microwave oven: power: 300 W; frequency: 2.45 GHz; temperature range: 60–250 °C [29]. The purity of the compounds was determined by a single spot on the TLC silica gel G plate. IR spectra were recorded on the Shimadzu IR convergence spectrometer using the KBr pellet method. NMR spectra (1H NMR and ^13^C NMR) are recorded on the Bruker Avance II 500 MHz NMR spectrometer. Mass spectra were performed on the JEOL GC mass spectrometer. The results in the spectrum head show that the molecular mass of the compounds produced was closer to the molecular mass of the expected compounds [30].

#### Chemistry

The comparative procedure (conventional as well as microwave irradiation method) of pyrazole derivatives by the synthetic route as shown in Scheme 1 is stated below in four steps and is already presented in our previous work with different substituents [28, 29].

Step 1: Synthesis of *N*'-(1-phenylethylidene) isonicotinohydrazide derivatives 1(A-C).
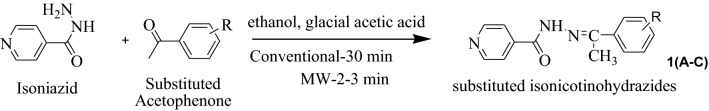


Whereas R = 4-NO_2_, 4-OCH_3_, 4-Br

**(Z)-*N*′-(1-(4-Nitrophenyl) ethylidene) isonicotinohydrazide (1A) **Yellow solid (71.6%); m.pt. 226–228 °C; IR (KBr, cm^−1^): 3280 (N–H str., CONH), 2900 (C-H str.), 1625 (C=C str.), 1590 (C=N str.), 1135 (C–N str.), 1657 (C=O str., CONH); ^1^H NMR (500 MHz, DMSO) δ (ppm) 9.06 (d, J = 6.3 Hz, 2H, CH, C_2_ & C_6_ of 4-pyridine), 7.96 (d, J = 6.3, 2H, CH, C_3_ & C_5_ of 4-pyridine), 7.36 (d, 2H, CH of C_2_ & C_6_ of 4-nitrophenyl), 8.24(d, 2H, CH of C_3_ & C_5_ of 4-nitrophenyl), 7.00 (s, NH of CONH).

**(Z)-*N*'-(1-(4-Methoxyphenyl) ethylidene) isonicotinohydrazide (1B)** Yellow solid (72%); m.pt. 193–195 °C; IR (KBr, cm^*−*1^): 3396 (N–H str., CONH), 2922 (C–H str.), 1625 (C=C str.), 1585 (C=N str.), 1150 (C–N str.), 1670 (C=O str., CONH); ^1^H NMR (500 MHz, DMSO) *δ* (ppm) 9.06 (d, J = 6.3 Hz, 2H, CH, C_2_ & C_6_ of 4-pyridine), 7.96 (d, J = 6.3, 2H, CH, C_3_& C_5_ of 4-pyridine), 7.00 (s, NH of CONH), 7.5 (d, J = 6.3 Hz, 2H, CH, C_2_ & C_6_ of 4-methoxyphenyl), 6.8 (d, J = 5.9 Hz, 2H, CH of C_3_ & C_5_ of 4-methoxyphenyl), 3.73 (s, 3H, OCH_3_).

**(Z)-*N'*-(1-(4-Bromophenyl) ethylidene) isonicotinohydrazide (1C)** Brown solid (89%); m.pt. 216–218 °C; IR (KBr, cm^*−*1^): 3380 (N–H str., CONH), 2918 (C-H str.), 1620 (C=C str.), 1590 (C=N str.), 1150 (C–N str.), 1690 (C=O str., CONH); ^1^H NMR (500 MHz, DMSO) *δ* (ppm) 9.06 (d, J = 6.3 Hz, 2H, CH, C_2_ & C_6_ of 4-pyridine), 7.96 (d, J = 6.3, 2H, CH, C_3_ & C_5_ of 4-pyridine), 7.00 (s, NH of CONH), 7.45 (d, J = 6.3 Hz, 2H, CH, C_2_ & C_6_ of 4-bromophenyl), 6.8 (d, J = 5.9 Hz, 2H, CH of C_3_ & C_5_ of 4-bromophenyl).

**Step 2: Synthesis of 1-isonicotinoyl-3-phenyl-4,5-dihydro-1** ***H*** **-pyrazole-4-carbaldehyde derivatives 2 (A–C)** 
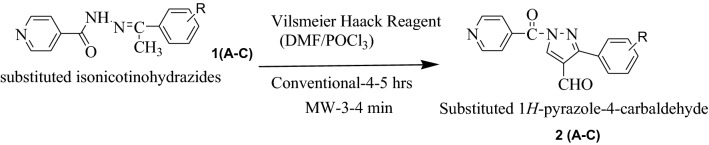


**3-(4-Nitrophenyl)-1-isonicotinoyl-1*H*-pyrazole-4-carbaldehyde (2A) **Yellow solid (70%); m.pt. 230–232 °C; IR (KBr, cm^*−*1^): 2900 (C–H str.), 1625 (C=C str.), 1590 (C=N str.), 1135 (C–N str.), 1657 (C=O str., CONH), 1558 (NO_2_ assym. str.), 1298 (NO_2_ symm. str.); ^1^H NMR (500 MHz, DMSO) *δ* (ppm) 9.06 (d, J = 6.3 Hz, 2H, CH, C_2_ & C_6_ of 4-pyridine), 7.96 (d, J = 6.3, 2H, CH, C_3_ & C_5_ of 4-pyridine), δ 7.5 (s, 1H, C_5_ of pyrazole), 7.36 (d, 2H, CH of C_2_ & C_6_ of 4-nitrophenyl), 8.24(d, 2H, CH of C_3_ & C_5_ of 4-nitrophenyl), 9.61 (s, 1H, CHO).

**3-(4-Methoxyphenyl)-1-isonicotinoyl-1*H*-pyrazole-4-carbaldehyde (2B) **Yellow solid (72%); mp 198–200 °C; IR (KBr, cm^*−*1^): 2980 (C–H str.), 1638 (C=C str.), 1586 (C=N str.), 1136 (C–N str.), 1670 (C=O str., CONH), 1236 (C–O–C asym. str.), 1095 (C–O–C sym. str.); ^1^H NMR (500 MHz, DMSO) *δ* (ppm) 9.06 (d, J = 6.3 Hz, 2H, CH, C_2_& C_6_ of 4-pyridine), 7.96 (d, J = 6.3, 2H, CH, C_3_ & C_5_ of 4-pyridine), δ 3.29 (s, 3H, OCH_3_), 7.5 (s, 1H, C-5 of pyrazole), 7.37 (d, 2H, CH of C_2_ & C_6_ of 4-methoxyphenyl), 6.83 (d, J = 2H, CH of C_3_ & C_5_ of 4-methoxyphenyl), 9.61 (s, 1H, CHO).

**3-(4-Bromophenyl)-1-isonicotinoyl-1*H*-pyrazole-4-carbaldehyde (2C) **Brown solid (85%); mp 222–224 °C; IR (KBr, cm^*−*1^):2996 (C-H str.), 1648 (C=C str.), 1580 (C=N str.), 1136 (C–N str.), 1672 (C=O str., CONH), 570 (C–Br str.); ^1^H NMR (500 MHz, DMSO) *δ* (ppm) 9.06 (d, J = 6.3 Hz, 2H, CH, C_2_ & C_6_ of 4-pyridine), 7.96 (d, J = 6.3, 2H, CH, C_3_ & C_5_ of 4-pyridine), 7.37 (d, 2H, CH of C_2_ & C_6_ of 4-bromophenyl), 7.49 (d, 2H, CH of C-3 & C-5 of 4-bromophenyl), 9.61 (s, 1H, CHO).

**Step 3: synthesis of (3-phenyl-4-((phenylimino) methyl)-4,5-dihydropyrazol-1-yl pyridin-4-yl)methanone derivatives/Schiff bases S (1–21).** 
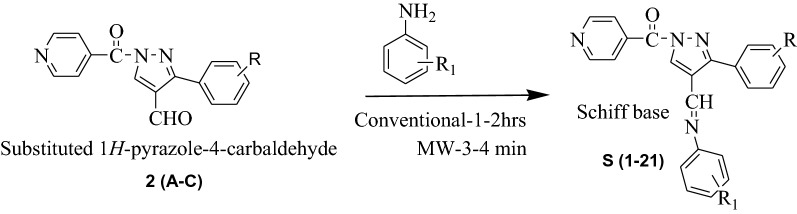


R_1_ = H, 4-NO_2_, 2-NO_2_, 4-Cl, 2-Cl, 4-OCH_3_, 2-OCH_3_, 4-Br.

**Step 4: Synthesis of 2-(1-isonicotinoyl-3-phenyl-4,5-dihydro-1*H*-pyrazol-4-yl)-3-phenyl thiazolidin-4-one derivatives P** (**1–21**).
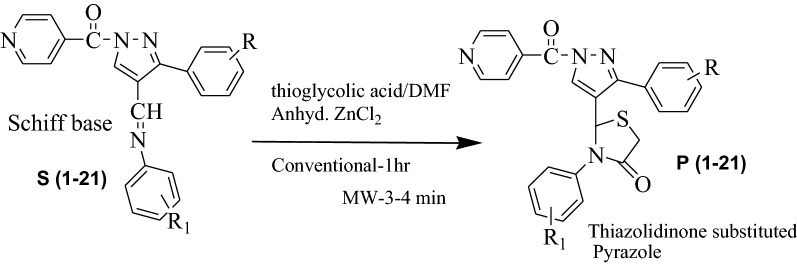


**2-(1-isonicotinoyl-3-(4-nitrophenyl)-1*H*-pyrazol-4-yl)-3-phenylthiazolidin-4-one (P-1) **Yellow solid (89.2%); m.pt- 244–246 °C; IIR (KBr, cm^−1^): 3200 (C-H str. Ar), 2450 (CH-S str.), 1680 (C = O str.pyrazole), 1730 (C = O str., thiazolidinone), 1361 (C = N str.), 1224 (Ar-N str.), 1157 (R–C-O str.), 1530 (NO_2_ asymm.str.), 1380 (NO_2_ symm.str.), 1200 (Ar-N str.), 1350 (C–C str.), 690–710 (Ar. CH-bend, monosubstituted).

**2-(1-isonicotinoyl-3-(4-nitrophenyl)-1*H*-pyrazol-4-yl)-3-(4-nitrophenyl)thiazolidin-4-one (P-2) **Yellow solid (87.4%); m.pt.- 266–268 °C; IR (KBr, cm^−1^): 3220 (C-H str. Ar), 2990 (CH-S str.), 1720 (C=O str., pyrazole),1733 (C=O str., thiazolidinone), 1310 (C=N str.), 1230 (Ar-N str.), 1157 (R–C-O str.), 1510 (NO_2_ asymm. str.), 1360 (NO_2_ symm. str.), 1270 (Ar-N str.), 1351 (C–C str.), 690–710 (Ar. CH-bend, monosubstituted).

**2-(1-isonicotinoyl-3-(4-nitrophenyl)-1*H*-pyrazol-4-yl)-3-(2-nitrophenyl)thiazolidin-4-one (P-3) **Yellow solid (83.1%); m.pt. 282–284 °C; IR (KBr, cm^−1^): 3220 (C–H str. Ar), 2990 (CH-S str.), 1720 (C=O str., pyrazole),1733 (C=O str., thiazolidinone), 1310 (C=N str.), 1230 (Ar-N str.), 1157 (R–C–O str.), 1510 (NO_2_ asymm. str.), 1360 (NO_2_ symm. str.), 1270 (Ar-N str.), 1351 (C–C str.), 690–710 (Ar. CH-bend, monosubstituted); ^1^H NMR (500 MHz, DMSO) δ 9.06 (d, J = 6.3 Hz, 2H, CH, C2 & C6 of 4-pyridine), 7.96 (d, J = 6.3 Hz, 2H, CH, C3 & C5 of 4-pyridine), 3.43 (s, J = 5.6 Hz, 2H, C-5 of thiazolidinone), 5.92 (d, 1H, CH, C_2_ of thiazolidinone), 7.3 (s, 1H, C_5_ of pyrazole), 7.36 (s, 1H, CH, C-6 of 2-nitrobenzene), 7.70 (s, 1H, CH, C-5 of 2-nitrobenzene), 7.50 (s, 1H, CH, C_4_ of 2-nitrobenzene), 8.24 (s, 1H, CH, C_3_ of 4-nitrobenzene).

**3-(4-chlorophenyl)-2-(1-isonicotinoyl-3-(4-nitrophenyl)-1*H*-pyrazol-4-yl)thiazolidin-4-one (P-4) **Creamy yellow (80%); m.pt.: 286–288 °C; IR (KBr, cm^−1^): 3201 (C-H str. Ar), 2928 (CH-S str.), 1680 (C = O str., pyrazole), 1736 (C=O str., thiazolidinone), 1361 (C=N str.), 1224 (Ar–N str.), 1157 (R–C–O str.), 1550 (NO_2_ asymm. str.), 1386 (NO_2_ symm. str.), 1298 (Ar–N str.), 1350 (C–C str.), 692 (C–Cl str.); ^1^H NMR (500 MHz, DMSO) δ 9.06 (d, J = 6.3 Hz, 2H, CH, C2& C6 of 4-pyridine), 7.96 (d, J = 6.3 Hz, 2H, CH, C_3_ & C_5_ of 4-pyridine), 3.43 (s, J = 5.6 Hz, 2H, C_5_ of thiazolidinone), 5.92 (d, 1H, CH, C_2_ of thiazolidinone), 7.3 (s, 1H, C_5_ of pyrazole), 4.6 (s, 1H, C_1_ of thiazolidinone), 7.5 (d, J = 4.5 Hz, 2H, CH of C_2_ & C_6_ of 4-chlorophenyl), 6.8 (d, J = 4.6 Hz, 2H, CH of C_3_ & C_5_ of 4-chlorophenyl), 7.38 (d, J = 4.8 Hz, 2H, CH, C_2_ & C_6_ of 2-nitrophenyl), 8.22 (d, J = 4.8 Hz, 2H, CH, C_3_ & C_5_ of 2-nitrophenyl.

**3-(3-chlorophenyl)-2-(1-isonicotinoyl-3-(4-nitrophenyl)-1*H*-pyrazol-4-yl)thiazolidin-4-one (P-5) **Creamy yellow (75.8%); m.pt. 274–276 °C; IR (KBr, cm^−1^): 3201 (C–H str. Ar), 2928 (CH–S str.), 1680 (C=O str., pyrazole), 1736 (C=O str., thiazolidinone), 1361 (C=N str.), 1224 (Ar–N str.), 1157 (R–C–O str.), 1550 (NO_2_ asymm. str.), 1386 (NO_2_ symm. str.), 1298 (Ar- N str.), 1350 (C–C str.), 692 (C–Cl str.).

**2-(1-isonicotinoyl-3-(4-nitrophenyl)-1*H*-pyrazol-4-yl)-3-(4-methoxyphenyl)thiazolidin-4-one (P-6) **Yellowish brown solid (72.6%); m.pt: 262–264 °C C; IR (KBr, cm^−1^): 3198 (C–H str. Ar), 2928 (CH–S str.), 1691 (C=O str., pyrazole),1726 (C = O str., thiazolidinone), 1361 (C=N str.), 1224 (Ar-N str.), 1157 (R–C–O str.), 1514 (NO_2_ asymm.str.), 1384 (NO_2_ symm.str.), 1296 (Ar-N str.), 1261 (C–O–C asymm. str.), 1076 (C–O–C symm. str.); ^1^H NMR (500 MHz, DMSO) δ 9.06 (d, J = 6.3 Hz, 2H, CH, C_2_ & C_6_ of 4-pyridine), 7.96 (d, J = 6.3 Hz, 2H, CH, C_3_ & C_5_ of 4-pyridine), 3.43 (s, J = 5.6 Hz, 2H, C_5_ of thiazolidinone), 5.92 (d, 1H, CH, C_2_ of thiazolidinone), 7.3 (s, 1H, C_5_ of pyrazole), 7.9 (d, J = 4.8 Hz, 2H, CH of C_2_ & C_6_ of 4-nitrophenyl), 8.2 (d, J = 4.8 Hz, 2H, CH of C_3_ & C_5_ of 4-nitrophenyl), 6.64 (s, 1H, CH, C_2_ of 4-methoxyphenyl), 6.70 (s, 1H, CH, C_4_ of 4-methoxyphenyl), 7.22 (s, 1H, CH, C_5_ of 4-methoxyphenyl), 6.60 (s, 1H, CH, C_6_ of 4-methoxyphenyl); ^13^C-NMR (CDCl_3_, 500 MHz, δppm): 149.8 (4-pyridine C_3_ & C_5_), 122.8 (4-pyridine C_2_ & C_6_), 167.0 (C=O), 151.8 (pyrazole C_3_), 46.1 (pyrazole C_4_), 71.1 (pyrazole C_5_), 140.0 (4-nitrophenyl C_1_), 130.0 (4-nitrophenyl C_2_ & C_6_), 121.0 (4-nitrophenyl C_3_ & C_5_), 60.7 (4-nitrophenyl C_4_), 47.8 (thiazolidin-4-one C_2_), 34 (thiazolidin-4-one C_5_), 170.9 (thiazolidin-4-one C_4_ (C=O)), 134 (4-methoxyphenyl C_1_), 122.6 (4-methoxyphenyl C_2_ & C_6_), 114 (4-methoxyphenyl C_3_ & C_5_), 156.0 (4-methoxyphenyl C_4_), 55.9 (O-CH_3_); m/z 503 (M^+^); Anal.: Calcd. for C_25_H_21_N_5_O_5_S: C, 59.638; H, 4.28; N, 13.918; S, 6.378; O, 15.898. Found: C, 59.276; H, 4.24; N, 13.886; S, 6.338; O, 15.890.

**2-(1-isonicotinoyl-3-(4-nitrophenyl)-1*H*-pyrazol-4-yl)-3-(2-methoxyphenyl)thiazolidin-4-one (P-7) **Yellowish brown solid (93.4%); m.pt.: 250–252 °C; IR (KBr, cm^−1^): 3198 (C–H str. Ar), 2928 (CH–S str.), 1691 (C=O str., pyrazole),1726 (C=O str., thiazolidinone), 1361 (C=N str.), 1224 (Ar-N str.), 1157 (R–C-O str.), 1514 (NO_2_ asymm.str.), 1384 (NO_2_ symm.str.), 1296 (Ar-N str.), 1261 (C–O–C asymm. str.), 1076 (C–O–C symm. str.)

**2-(1-isonicotinoyl-3-(4-methoxyphenyl)-1*H*-pyrazol-4-yl)-3-phenylthiazolidin-4-one (P-8) **Yellow solid (82.5%); m.pt. 300–302 °C; IR (KBr, cm^−1^): 3252 (C–H str. Ar), 2932 (CH–S str.), 1688 (C=O str., pyrazole),1740 (C=O str., thiazolidinone), 1548 (C=N str.), 1226 (Ar–N str.), 1160 (R–C=O str.), 1338 (C=N str.), 1330 (C–O–C asymm. str.), 1128 (C–O–C symm. str.).

**2-(1-isonicotinoyl-3-(4-methoxyphenyl)-1*H*-pyrazol-4-yl)-3-(4-nitrophenyl)thiazolidin-4-one (P-9) **Yellow solid (76.3%); m.pt. 308–310 °C; IIR (KBr, cm^−1^): 3152 (C–H str. Ar), 2928 (CH-S str.), 1681 (C=O str., pyrazole),1733 (C=O str., thiazolidinone), 1649 (C=N str.), 1226 (Ar-N str.), 1157 (R–C=O str.), 1338 (C=N str.), 1514 (NO_2_ asymm.str.), 1384 (NO_2_ symm.str.), 1298 (C–O–C asymm. str.), 1108 (C–O–C symm. str.).

**2-(1-isonicotinoyl-3-(4-methoxyphenyl)-1*H*-pyrazol-4-yl)-3-(2-nitrophenyl)thiazolidin-4-one (P-10) **Yellow white solid (64.7%); m.pt. 322–324 °C; IR (KBr, cm^−1^): 3137 (C–H str. Ar), 2962 (CH–S str.), 1670 (C=O str., pyrazole), 1735 (C=O str., thiazolidinone), 1617 (C=N str.), 1284 (Ar-N str.), 1175 (R–C–O str.), 1319, 1411 (C=N str.), 1520 (NO_2_ asymm. str.), 1350 (NO_2_ symm. str.), 1310 (C–O–C asymm. str.), 1198 (C–O–C symm. str.); ^1^H NMR (500 MHz, DMSO) δ 9.06 (d, J = 6.3 Hz, 2H, CH, C_2_ & C_6_ of 4-pyridine), 7.96 (d, J = 6.3 Hz, 2H, CH, C_3_ & C_5_ of 4-pyridine), 33.43 (s, J = 5.6 Hz, 2H, C_5_ of thiazolidinone), 5.92 (d, 1H, CH, C_2_ of thiazolidinone), 7.3 (s, 1H, C_5_ of pyrazole), 7.9 (d, J = 4.8 Hz, 2H, CH of C_2_ & C_6_ of 4-nitrophenyl), 8.2 (d, J = 4.8 Hz, 2H, CH of C_3_ & C_5_ of 4-nitrophenyl), 8.24 (s, 1H, CH, C_3_ of 2-nitrophenyl), 7.50 (s, 1H, CH, C_4_ of 2-nitrophenyl), 7.70 (s, 1H, CH, C_5_ of 2-nitrophenyl), 7.36 (s, 1H, CH, C_6_ of 2-nitrophenyl).

**3-(4-chlorophenyl)-2-(1-isonicotinoyl-3-(4-methoxyphenyl)-1*H*-pyrazol-4-yl)thiazolidin-4-one (P-11) **Yellowish white (68.8%); m.pt. 318–320 °C; IR (KBr, cm^−1^): 3137 (C–H str. Ar), 2959 (CH–S str.), 1681 (C=O str., pyrazole), 1740 (C=O str., thiazolidinone), 1588 (C=N str.), 1221 (Ar-N str.), 1157 (R–C–O str.), 1317, 1415 (C=N str.), 696 (C–Cl str.), 1330 (C–O–C asymm. str.), 1180 (C–O–C symm. str.); ^1^H NMR (500 MHz, DMSO) δ 9.06 (d, J = 6.3, 2H, CH, C_2_ & C_6_ of 4-pyridine), 7.96 (d, J = 6.3, 2H, CH, C_3_ & C_5_ of 4-pyridine), 3.43 (s, J = 5.6 Hz, 2H, C_5_ of thiazolidinone), 5.92 (d, 1H, CH, C_2_ of thiazolidinone), 7.3 (s, 1H, C_5_ of pyrazole), 6.64 (s, 1H, CH, C_2_ of 4-methoxyphenyl), 6.70 (s, 1H, CH, C_4_ of 4-methoxyphenyl), 7.22 (s, 1H, CH, C_5_ of 4-methoxyphenyl), 6.60 (s, 1H, CH, C_6_ of 4-methoxyphenyl),7.04 (d, J = 5.9 Hz, 2H, CH, C_2_ & C_6_ of 4-chlorophenyl), 7.32 (d, J = 5.9 Hz, 2H, CH, C_3_ & C_5_ of 4-chlorophenyl).

**3-(3-chlorophenyl)-2-(1-isonicotinoyl-3-(4-methoxyphenyl)-1*H*-pyrazol-4-yl)thiazolidin-4-one (P-12) **Yellowish white solid (72.4%); m.pt. 338–340 °C; IR (KBr, cm^−1^): 3189 (C–H str., Ar), 2919 (CH–S str.), 1669 (C=O str., pyrazole), 1733 (C=O str., thiazolidinone), 1556 (C=N str.), 1222 (Ar-N str.), 1140 (R–C–O str.), 678 (C–Cl str.), 1310 (C–O–C asymm. str.), 1198 (C–O–C symm. str.); ^1^H NMR (500 MHz, DMSO) δ 9.06 (d, J = 6.3 Hz, 2H, CH, C_2_ & C_6_ of 4-pyridine), 7.96 (d, J = 6.3 Hz, 2H, CH, C_3_ & C_5_ of 4-pyridine), 3.43 (s, J = 5.6 Hz, 2H, C_5_ of thiazolidinone), 5.92 (d, 1H, CH, C_2_ of thiazolidinone), 7.3 (s, 1H, C_5_ of pyrazole), 6.61 (s, 1H, CH, C_2_ of 4methoxyphenyl), 6.75 (s, 1H, CH, C_4_ of 4-methoxyphenyl), 7.20 (s, 1H, CH, C_5_ of 4-methoxyphenyl), 6.66 (s, 1H, CH, C_6_ of 4-methoxyphenyl), 3.73 (s, 3H, OCH_3_), 7.11 (s, 1H, CH, C_2_ of 3-chlorophenyl, 7.25 (d, J = 5.7 Hz, 2H, CH, C_4_ & C_5_ of 3-chlorophenyl), 6.98 (s, 1H, CH, C_6_ of 3-chlorophenyl); ^13^C-NMR (CDCl_3_, 500 MHz, δppm): 149.8 (4-pyridine C_3_ & C_5_), 122.8 (4-pyridine C_2_ & C_6_), 167.0 (C=O), 151.8 (pyrazole C_3_), 46.1 (pyrazole C_4_), 71.1 (pyrazole C_6_), 126.0 (4-methoxyphenyl C_1_), 130.0 (4-methoxyphenyl C_2_ & C_6_), 114.0 (4-methoxyphenyl C_3_ & C_5_), 163 (4-methoxyphenyl C_4_), 55.9 (O-CH_3_), 47.8 (thiazolidin-4-one C_2_), 34 (thiazolidin-4-one C_5_), 170.9 (thiazolidin-4-one C_4_ (C=O)), 143 (3-chlorophenyl C_1_), 122.6 (3-chlorophenyl C_2_ & C_6_), 134.5 (3-chlorophenyl C_3_), 124.0 (3-chlorophenyl C_4_), 130.4 (3-chlorophenyl C_5_); LC–MS: m/z 490 (M^+^); Anal.: Calcd. for C_25_H_19_ClN_4_O_3_S Anal. Calcd.: C, 61.168; H, 3.98; N, 11.418; S, 6.538; Cl, 7.228; O, 9.788. Found: C, 61.068; H, 3.68; N, 11.218; S, 6.508; Cl, 7.218; O, 9.748.

**2-(1-isonicotinoyl-3-(4-methoxyphenyl)-1*H*-pyrazol-4-yl)-3-(4-methoxyphenyl) thiazolidin-4-one (P-13) **Yellow solid (76.6%); m.pt. 320–322 °C; IR (KBr, cm^−1^): 3354 (C–H str. Ar), 2964 (CH–S str.), 1690 (C=O str., pyrazole),1722 (C=O str., thiazolidinone), 1593 (C=N str.), 1259 (Ar-N str.), 1174 (R–C–O str.), 1338, 1417 (C=N str.);1292 (C–O–C asymm. str.), 1126 (C–O–C symm. str.); ^1^H NMR (500 MHz, DMSO) δ 9.06 (d, J = 6.3 Hz, 2H, CH, C_2_ & C_6_ of 4-pyridine), 7.96 (d, J = 6.3 Hz, 2H, CH, C_3_ & C_5_ of 4-pyridine), 3.43 (s, J = 5.6 Hz, 2H, C_5_ of thiazolidinone), 5.92 (d, 1H, CH, C_2_ of thiazolidinone), 7.3 (s, 1H, C_5_ of pyrazole), 6.66 (s, 1H, CH, C_2_ of 4-methoxyphenyl), 6.70 (s, 1H, CH, C_4_ of 4-methoxyphenyl), 7.24 (s, 1H, CH, C_5_ of 4-methoxyphenyl), 6.69 (s, 1H, CH, C_6_ of 4-methoxyphenyl), 7.20 (s, 1H, CH, C_5_); ^13^C-NMR (CDCl_3_, 500 MHz, δppm): 149.8 (4-pyridine C_3_ & C_5_), 122.8 ( 4-pyridine C_2_ & C_6_), 167.0 (C=O), 151.8 (pyrazole C_3_), 46.1 (pyrazole C_4_), 71.1 (pyrazole C_6_), 126.0 (4-methoxyphenyl C_1_), 130.0 (4-methoxyphenyl C_2_ & C_6_), 114.0 (4-methoxyphenyl C_3_ & C_5_), 163.0 (4-methoxyphenyl C_4_), 55.9 (O–CH_3_), 47.8 (thiazolidin-4-one C_2_), 34 (thiazolidin-4-one C_5_), 170.9 (thiazolidin-4-one C_4_ (C=O)), 134 (4-methoxyphenyl C_1_), 122.6 (4-methoxyphenyl C_2_ & C_6_), 114 (4-methoxyphenyl C_3_ & C_5_), 156.0 (4-methoxyphenyl C_4_), 55.9 (O-CH_3_).; LC–MS: m/z 486 (M^+^); Anal.: Calcd. for C_26_H_22_N_4_O_4_S C, 64.188; H, 4.568; N, 11.528; S, 6.598; O, 13.158; Found: C, 63.752; H, 4.576; N, 11.273; S, 6.320; O, 13.614..

**2-(1-isonicotinoyl-3-(4-methoxyphenyl)-4,5-dihydro-1*H*-pyrazol-4-yl)-3-(2-methoxyphenyl)thiazolidin-4-one (P-14)** Yellow solid (84.3%); m.pt. 336–338 °C; IR (KBr, cm^−1^): 3217 (C–H str. Ar), 2968 (CH–S str.), 1681 (C=O str., pyrazole), 1735 (C=O str., thiazolidinone), 1645 (C=N str.), 1257 (Ar–N str.), 1174 (R–C-O str.), 1338, 1415 (C=N str.), 1310 (C–O–C asymm. str.), 1120 (C–O–C symm. str.); ^1^H NMR (500 MHz, DMSO) δ 9.06 (d, J = 6.3 Hz, 2H, CH, C_2_ & C_6_ of 4-pyridine), 7.96 (d, J = 6.3 Hz, 2H, CH, C_3_ & C_5_ of 4-pyridine), 33.43 (s, J = 5.6 Hz, 2H, C_5_ of thiazolidinone), 5.92 (d, 1H, CH, C_2_ of thiazolidinone), 7.3 (s, 1H, C_5_ of pyrazole), 6.82 (s, 1H, CH, C_3_ of 2-methoxyphenyl), 7.13 (s, 1H, CH, C_4_ of 2-methoxyphenyl), 7.87 (s, 1H, CH, C_5_ of 2-methoxyphenyl), 6.99 (s, 1H, CH, C_6_ of 2-methoxyphenyl), 3.73 (s, 3H, OCH_3_).

**2-(3-(4-bromophenyl)-1-isonicotinoyl-1*H*-pyrazol-4-yl)-3-phenylthiazolidin-4-one (P-15) **Brown solid (84.7%); m.pt. 256–258 °C; IR (KBr, cm^−1^): 3398 (C–H str. Ar), 2848 (CH–S str.), 1680 (C=O str., pyrazole), 1730 (C=O str., thiazolidinone), 1649 (C=N str.), 1226 (Ar-N str.), 1198 (R–C-O str.), 1420 (C=N str.), 560 (C–Br str.); ^1^H NMR (500 MHz, DMSO) δ 9.06 (d, J = 6.3 Hz, 2H, CH, C_2_ & C_6_ of 4-pyridine), 7.96 (d, J = 6.3 Hz, 2H, CH, C_3_ & C_5_ of 4-pyridine), 33.43 (s, J = 5.6 Hz, 2H, C_5_ of thiazolidinone), 5.92 (d, 1H, CH, C_2_ of thiazolidinone), 7.3 (s, 1H, C_5_ of pyrazole),7.10 (d, J = 4.5 Hz, 2H, CH, C_2_ & C_6_ of bromo phenyl ring), 7.31 (d, J = 4.7 Hz, 2H, CH, C_3_ & C_5_ of bromophenyl ring), 7.24 (m, 1H, C_4_ of bromophenyl).

**2-(3-(4-bromophenyl)-1-isonicotinoyl-1*H*-pyrazol-4-yl)-3-(4-nitrophenyl)thiazolidin-4-one (P-16) **Yellowish brown solid (68.9%); m.pt. 268–270 °C; IR (KBr, cm^−1^): 3398(C-H str. Ar), 2900 (CH-S str.), 1690 (C=O str., pyrazole),1740 (C=O str., thiazolidinone), 1649 (C=N str.), 1229 (Ar–N str.), 1154 (R–C–O str.), 1418 (C=N str.); 1520 (NO_2_ asymm. str.), 1310 (NO_2_ symm. str.), 1150 (Ar–N str.), 570 (C–Br str.).

**2-(3-(4-bromophenyl)-1-isonicotinoyl-4,5-dihydro-1*H*-pyrazol-4-yl)-3-(2-nitrophenyl) thiazolidin-4-one (P-17) **Brown solid (72.0%); m.pt. 252–254 °C; IR (KBr, cm^−1^): 3200 (C–H str. Ar), 2850 (CH–S str.), 1690 (C=O str., pyrazole), 1735 (C=O str., thiazolidinone), 1649 (C=N str.), 1226 (Ar-N str.), 1157 (R–C–O str.), 1418 (C=N str.); 1518 (NO_2_ asymm. str.), 1350 (NO_2_ symm. str.), 1198 (Ar-N str.), 580 (C–Br str.).

**2-(3-(4-bromophenyl)-1-isonicotinoyl-1*H*-pyrazol-4-yl)-3-(4-chlorophenyl)thiazolidin-4-one (P-18) **Brown solid (79.8%); m.pt. 244–246 °C; IIR (KBr, cm^−1^): 3215 (C–H str. Ar), 2936 (CH–S str.), 1680 (C=O str., pyrazole), 1735 (C=O str., thiazolidinone), 1560 (C=N str.), 1228 (Ar–N str.), 1157 (R–C–O str.), 1414 (C=N str.); 690 (C–Cl str.), 578 (C–Br str.); ^1^H NMR (500 MHz, DMSO) δ 9.06 (d, J = 6.3 Hz, 2H, CH, C_2_ & C_6_ of 4-pyridine), 7.96 (d, J = 6.3 Hz, 2H, CH, C_3_ & C_5_ of 4-pyridine), 33.43 (s, J = 5.6 Hz, 2H, C_5_ of thiazolidinone), 5.92 (d, 1H, CH, C_2_ of thiazolidinone), 7.3 (s, 1H, C_5_ of pyrazole), 7.5 (m, 4H, CH of C_2_, C_3_, C_5_ & C_6_ of 4-bromophenyl),7.04 (d, J = 4.6 Hz, 2H, CH, C_2_ & C_6_ of 4-chlorophenyl), 7.32 (d, J = 4.6 Hz, 2H, CH, C_3_ & C_5_ of 4- chlorophenyl).

**2-(3-(4-bromophenyl)-1-isonicotinoyl-4,5-dihydro-1*H*-pyrazol-4-yl)-3-(3-chlorophenyl)thiazolidin-4-one (P-19) **Brown solid (72.3%); m.pt. 248–250 °C; IR (KBr, cm^−1^): 3125 (C–H str. Ar), 2910 (CH–S str.), 1670 (C=O str., pyrazole), 1735 (C=O str., thiazolidinone), 1589 (C=N str.), 1224 (Ar–N str.), 1150 (R–C-O str.), 1410 (C=N str.); 694 (C–Cl str.), 570 (C–Br str.).

**2-(3-(4-bromophenyl)-1-isonicotinoyl-1*H*-pyrazol-4-yl)-3-(4-methoxyphenyl) thiazolidin-4-one (P-20) **Brown solid (83.2%); m.pt. 252–254 °C; IR (KBr, cm^−1^): 3120 (C-H str. Ar), 2924 (CH-S str.), 1669 (C=O str., pyrazole), 1740 (C=O str., thiazolidinone), 1589 (C=N str.), 1226 (Ar–N str.), 1159 (R–C–O str.), 1340, 1415 (C=N str.); 570 (C–Br str.), 1317 (asymm. C–O–C str.), 1090 (symm. C–O–C str.); ^1^H NMR (500 MHz, DMSO): δ 9.06 (d, J = 6.3 Hz, 2H, CH, C_2_ & C_6_ of 4-pyridine), 7.96 (d, J = 6.3 Hz, 2H, CH, C_3_ & C_5_ of 4-pyridine), 3.43 (s, J = 5.6 Hz, 2H, C_5_ of thiazolidinone), 5.92 (d, 1H, CH, C_2_ of thiazolidinone), 7.3 (s, 1H, C_5_ of pyrazole), 7.5 (m, 4H, CH of C_2_, C_3_, C_5_ & C_6_ of 4-bromophenyl), 6.61 (s, 1H, CH, C_2_ of 4-methoxyphenyl), 6.75 (s, 1H, CH, C_4_ of 4-methoxyphenyl), 7.20 (s, 1H, CH, C_5_ of 4-methoxyphenyl), 6.66 (s, 1H, CH, C_6_ of 3-methoxyphenyl), 3.73 (s, 3H, OCH_3_); ^13^C-NMR (CDCl_3_, 500 MHz, δppm): 149.8 (4-pyridine C_3_ & C_5_), 122.8 (4-pyridine C_2_ & C_6_), 167.0 (C=O), 151.8 (pyrazole C_3_), 46.1 (pyrazole C_4_), 71.1 (pyrazole C_6_), 133.0.0 (4-bromophenyl C_1_), 131.0 (4-bromophenyl C_2_ & C_6_), 131.0 (4-bromophenyl C_3_ & C_5_), 125.4 (4-bromophenyl C_4_), 47.8 (thiazolidin-4-one C_2_), 34 (thiazolidin-4-one C_5_), 170.9 (thiazolidin-4-one C_4_ (C=O)), 134 (4-methoxyphenyl C_1_), 122.6 (4-methoxyphenyl C_2_ & C_6_), 114 (4-methoxyphenyl C_3_ & C_5_), 156.0 (4-methoxyphenyl C_4_), 55.9 (O-CH_3_); LC–MS: m/z 535 (M^+^); Anal.: Calcd. for C_25_H_19_BrN_4_O_3_S: C, 56.088; H, 3.588; Br, 14.928; N, 10.468; S, 5.998; O, 8.968. Found: C, 56.078; H, 3.548; Br, 14.878; N, 10.438; S, 5.978; O, 8.938.

**2-(3-(4-bromophenyl)-1-isonicotinoyl-4,5-dihydro-1*H*-pyrazol-4-yl)-3-(2-methoxy phenyl) thiazolidin-4-one (P-21) **Yellowish brown solid (69.1%); m.pt. 260–262 °C; IR (KBr, cm^−1^): 3337 (C–H str., Ar), 2924 (CH–S str.), 1670 (C=O str., pyrazole), 1710 (C=O str., thiazolidinone), 1549 (C=N str.), 1225 (Ar–N str.), 1157 (R–C-O str.), 1338, 1415 (C=N str.), 590 (C–Br str.), 1311 (asymm. C–O–C str.), 1095 (symm. C–O–C str.); ^1^H NMR (500 MHz, DMSO) δ 9.06 (d, J = 6.3 Hz, 2H, CH, C_2_ & C_6_ of 4-pyridine), 7.96 (d, J = 6.3 Hz, 2H, CH, C_3_ & C_5_ of 4-pyridine), 3.43 (s, J = 5.6 Hz, 2H, C_5_ of thiazolidinone), 5.92 (d, 1H, CH, C_2_ of thiazolidinone), 7.3 (s, 1H, C_5_ of pyrazole), 7.5 (m, 4H, CH of C_2_, C_3_, C_5_ & C_6_ of 4-bromophenyl), 5.59 (s, 1H, CH, C_2_ of 2-methoxyphenyl), 6.25 (s, 1H, CH, C_4_ of 2-methoxyphenyl), 7.18 (s, 1H, CH, C_5_ of 2-methoxyphenyl), 6.66 (s, 1H, CH, C_6_ of 2-methoxyphenyl), 3.73 (s, 3H, OCH_3_); ^13^C-NMR (CDCl_3_, 500 MHz, δppm): 149.8 (4-pyridine C_3_ & C_5_), 122.8 ( 4-pyridine C_2_ & C_6_), 167.0 (C=O), 151.8 (pyrazole C_3_), 46.1 (pyrazole C_4_), 71.1 (pyrazole C-6), 133.0.0 (4-bromophenyl C_1_), 131.0 (4-bromophenyl C_2_ & C_6_), 131.0 (4-bromophenyl C_3_ & C_5_), 125.4 (4-bromophenyl C_4_), 47.8 (thiazolidin-4-one C_2_), 34 (thiazolidin-4-one C_5_), 170.9 (thiazolidin-4-one C_4_ (C=O)), 134 (4-methoxyphenyl C_1_), 120.4 (2-methoxyphenyl C_2_ & C_6_), 114 (4-methoxyphenyl C_3_ & C_5_), 152.0 (2-methoxyphenyl C_4_), 55.9 (O-CH_3_); LC–MS: m/z 535 (M^+^); Anal.: Calcd. for C_25_H_19_BrN_4_O_3_S: C, 56.088; H, 3.588; Br, 14.928; N, 10.468; S, 5.998; O, 8.968. Found: C, 56.088; H, 3.548; Br, 14.878; N, 10.438; S, 5.978; O, 8.938.

### Biological evaluation

#### In vitro cytotoxicity assay/cell viability assay

**Fig. 5:**
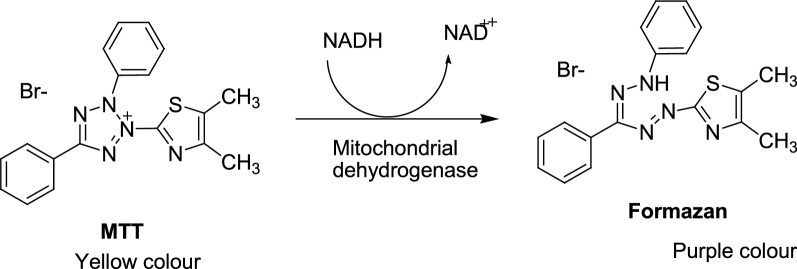
Systematic conversion of MTT to formazan [34]

When investigating a new drug, whether natural or synthesized, we should examine its safety for the host cell or the cytotoxic effect on the cancer cell. This is illustrious as the cell viability test. There are several methods to determine cell viability, among which the MTT test is a frequently used method. The MTT test was carried out using the 3-(4,5-dimethyl-2-thiazolyl)-2,5-diphenyl-2*H*-tetrazolium (MTT) colorimetric test [33, 34]. MTT is the frequently used method to assess cell viability and cytotoxicity for drug detection. The MTT test based on the reduction of MTT (yellow color) depends on cellular metabolic activities due to H-dependent cellular NAD (P) oxidoreductase enzymes [33]. Healthy fast-growing cells show high rates of reduction of MTT to formazan while dead or inactive cells do not. The final product of MTT reduction is a purple formazan that can be easily dissolved in DMSO. The high intensity of the purple color indicates greater cell viability, while a decrease in the intensity of the purple color means a reduced number of cells and, therefore, the cytotoxicity of the given substance. The systematic conversion of MTT to formazan is illustrated in Fig. 5 [34].

#### Procedure [33, 34]

**Fig. 6:**
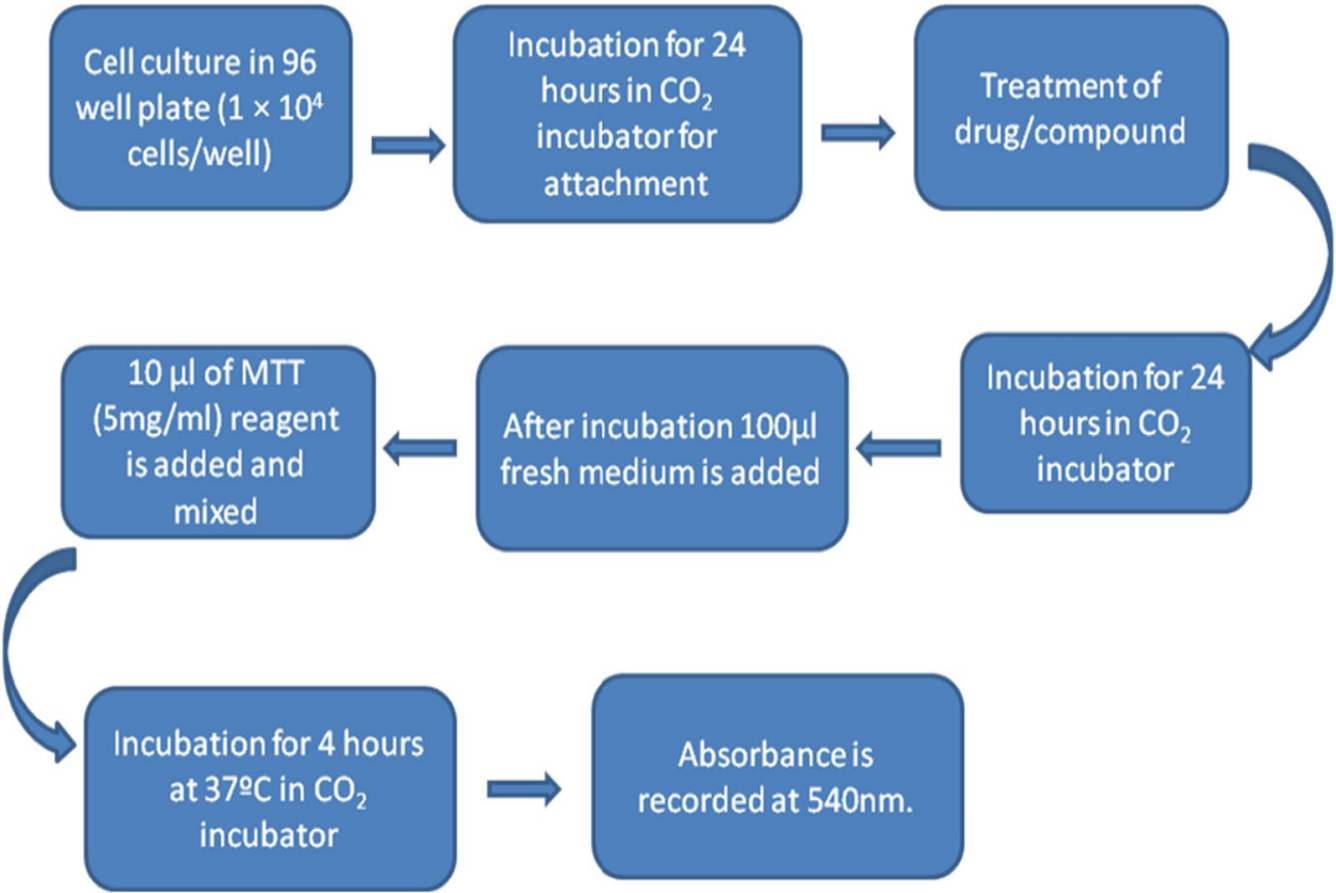
Schematic representation of MTT assay protocol

In the present study, systematic experimental steps as shown in Fig. 6 to determine the potential cytotoxicity of the drug at different concentrations using the MTT assay. The decrease in absorbance at 540 nm is shown in cells treated with an increasing drug concentration compared to control cells without any treatment. A decrease in absorbance in cells treated with drugs suggests cytotoxicity. The MTT test considerably helps investigators to determine whether one of the compounds to be tested has cell toxicity or antiproliferative activity [33, 34].

To test the anticancer activities of the synthesized compounds (selected based on docking studies), we evaluated the antiproliferative activity of compounds **P (1–21)** against HCT116 and MCF-7 cell lines. All the experiments were repeated at least thrice independently, the 50% inhibitory concentration (IC_50_) of new hybrids was determined (as the anticancer drug concentration causing a 50% reduction in cell viability) and calculated using a trendline equation. IC_50_ values of the new hybrids in μM against various cell lines are given in Table 2.

#### Aurora A kinase inhibition assay

The assay was carried out by the following method:

At 30 °C, dilute enzyme (aurora A), substrates (NADH, NaCl, MgCl_2_, PEP, LDH, PK, and histones H3), ATP, and inhibitors (selected compounds as per in silico studies) in kinase buffer (HEPES) and this to 96 well plates and incubate for 60 min. Once the ATP substrate was added, the reaction began. With the reaction, NADH was continuously transformed into NAD^+^ The activity of Aurora-A can be assayed by measuring the consumption of nicotinamide adenine dinucleotide plus hydrogen (NADH) at 340 nm. Absorbance at 340 nm was continuously recorded for 10 min. Inhibition of recombinant human Aurora-A by inhibitors was initially screened via an enzyme-coupled continuous spectrophotometric assay (Kishore et al., 2008; Aixia et al., 2011; Fancelli D et al., 2006). The aurora kinase inhibitor, VX-680 (Tozasertib) (Duong et al., 2016) was used as the positive control [12, 13, 20, 21, 36].

The pyrazole derivatives **P (1–21)** were examined for their capability to inhibit the activity of Aurora A kinase (Table 2) to clarify their structure–activity relationships [22, 23]. The IC_50_ value of compounds was calculated by using the trendline equation as shown in Table 2.

### Docking studies

To study the binding mode and explore the molecular interactions between the ligands and protein, docking studies were carried out for the synthesized pyrazole derivatives with the saluted co-crystallized structure of Aurora-A Kinase by using AutoDock Vina and the graphical user interface, AutoDock Tools (ADT) installed on Windows-7 [38].

Following protocol was followed for the docking studies:

*Ligand preparation* The 2D structures of the designed pyrazole derivatives were drawn by using Chem Sketch (ACDLABS 12.0) and stored in a database in SDF structure format [39]. All the structures were converted to 3D structures with the help of 3D optimization tool i.e., Ligprep, and storing them as a PDBQT file.

*Protein preparation* The X-ray co-crystal structure of Aurora-A Kinase protein was obtained from the RCSB protein data bank (http://www.rcsb.org/pdb). After evaluating the numbers of entries, the best protein (PDB entry 2bmc) [25, 37] was selected by analyzing all the proteins and choosing one with the highest resolution i.e., 2.00 A The PDB file of Aurora Kinase A was abridged with the help of PyMOL and α chain was removed along with the complexed inhibitor. All water molecules and interacting ions were removed (The PyMOL Molecular Graphics System). The PDBQT file for the protein was generated with the help of Auto Dock Tools by the addition of all polar hydrogen atoms followed by charge assignment to the macromolecule.

*Validation of docking protocol in glide* The most suitable method of evaluating the accuracy of the docking procedure is to determine how closely the lowest energy pose predicted by the scoring function resembles an experimental binding mode as determined by X-ray crystallography.

*Generation of grid and ligand docking* Docking studies on designed derivatives prepared through Ligprep were carried out in the active site of the protein. The calculations of grid parameters were accomplished by the Grid tool in ADT. The grid parameter files possessing all the information about the size of grid, protein, ligand, and geometry of search space were prepared and saved as ‘Conf.txt’. The optimized ligand molecules in PDBQT format were docked in the active site of Aurora Kinase A with Auto Dock Vina. Docking runs were launched from the command line, followed by the generation and scoring of best poses, for every ligand using the scoring function. At the end of the docking, ligands with the most favorable free energy of binding were selected. The protein–ligand interactions were further analyzed for the docked ligands by using PyMOL and the best poses in the binding site were drawn [38, 39].

## Data Availability

We have presented all our main data in the form of tables and figures.

## Abbreviations

Anal. Calcd.: Analytically calculated
CDCl3: Deuterated chloroform
cm: Centimetre
DMF: Dimethylformamide
DMSO: Dimethylsulfoxide
HCT: Human colorectal
H-NMR: Proton nuclear magnetic resonance
IC_50_: Half maximal inhibitory concentration
IR: Infrared Spectroscopy
LC–MS: Liquid Chromatography Mass Spectrometry
MCF: Michigan Cancer Foundation
m.pt.: Melting Point
μM: Micro mole
MW: Molecular weight
MTT: 3-(4,5-Dimethylthiazol-2-yl)-2,5-diphenyl tetrazolium bromide
PDB: Protein data bank
Rf: Retention factor
ppm: Parts Per Million
Str.: Stretching
TZD: Thiazolidin-2,4-dione
